# Causal role of immune cells in uveitis: Mendelian randomization study

**DOI:** 10.3389/fimmu.2024.1402074

**Published:** 2024-07-09

**Authors:** Jiahui Wu, Caocao Fang, Yongwei Zhou, Menghua Wang, Qiuming Li, Shuqian Dong

**Affiliations:** Department of Ophthalmology, The First Affiliated Hospital of Zhengzhou University, Henan Provincial Ophthalmic Hospital, Zhengzhou, China

**Keywords:** uveitis, immune cells, Mendelian randomization, causal association, SNP

## Abstract

**Background:**

Uveitis, characterized by inflammation of the iris, ciliary body, and choroid, presents a significant global clinical challenge, contributing substantially to visual impairment. Risk factors include autoimmune diseases and immune cell dysfunctions, yet many remain unidentified. Immune cells, notably T cells, B cells, and monocytes, play pivotal roles in uveitis pathogenesis. While biologic agents show promise, comprehensive studies on immune cell types in ocular diseases are lacking. Genome-wide association studies (GWAS) and Mendelian randomization (MR) present promising avenues to elucidate genetic susceptibilities and causal relationships between immune cell traits and uveitis risk.

**Methods:**

Two-sample MR analysis was used to evaluate the causal relationship between 731 immune cells and uveitis, and genome-wide significance analysis was performed for genetic variation in 731 immune cells traits (P < 5 × 10^-8^). Immune characteristics include median fluorescence intensity (MFI), relative cell counts (RC), absolute cell counts (AC), and morphological parameters (MP), which were determined by published GWAS, and public data from the IEU Open GWAS database. The main analysis method of MR is inverse variance weighting (IVW). Heterogeneity and horizontal pleiotropy were also assessed.

**Results:**

5 immunophenotypes, including CD62L-DC %DC, IgD+ CD38^dim^ %B cell, CD3 on CM CD4+T cell, CD3 on CD45RA-CD4 +T cell, and CD3 on CD39+ CD4+ Treg may increase the risk of uveitis. 5 immunophenotypes, including CD11b on CD33^dim^ HLA DR-Myeloid cell, HLA DR on CD33^dim^ HLA DR+ CD11b-myeloid cell, CD14-CD16 + %monocyte, HLA DR on CD14-CD16 + monocyte and PDL-1 on CD14-CD16 + monocyte was negatively associated with the risk of uveitis. Among them, HLA DR on CD14-CD16 + monocyte (OR=0.921, 95%CI =0.875-0.970, *P*=0.001) and HLA DR on CD33^dim^ HLA DR+ CD11b- (OR=0.879, 95%CI = 0.833-0.927, *P*=0.00) were negatively associated with the risk of uveitis in bi-direction.

**Conclusion:**

These results indicate that 10 immune cells traits are significantly associated with the risk of developing uveitis and 2 of them were strongly associated with uveitis bi-directionally, after excluding the effects of confounding factors such as some immune diseases, which provided new ideas and therapeutic targets for the study of immune mechanism of uveitis.

## Introduction

Uveitis, an inflammation affecting the iris, ciliary body, and choroid, poses a substantial clinical challenge. This condition significantly contributes to global visual impairment, with around 5-10% of cases worldwide, and approximately 35% leading to severe vision loss or blindness (1). Risk factors for uveitis include autoimmune diseases, such as ankylosing spondylitis, and functional impairments in immune cells, such as Tregs (2). However, many additional immune risk factors remain unidentified. Therefore, investigating the causal relationships between a broad spectrum of immune cells and the risk of uveitis could significantly improve the identification of high-risk populations, enabling early intervention. Moreover, this approach can provide critical insights for the research and development of targeted therapeutic strategies (3).

It is widely acknowledged that immune cells play a significant role in the pathogenesis of uveitis. For instance, T cells, B cells, and monocytes play crucial roles in uveitis, with a particular emphasis on T helper cells (Th1 and Th17) closely associated with the onset and progression of the disease (4). Consequently, therapeutic strategies targeting immune cells and their markers are continuously evolving. Among these, biologics such as anti-TNF-α, anti-IL-6, and anti-IL-17 monoclonal antibodies have demonstrated promising efficacy in modulating immune responses (5, 6). For example, the use of biologics like adalimumab, an anti-TNF-α agent, effectively alleviates symptoms of uveitis and significantly reduces recurrence rates (7, 8). Additionally, research has identified specific immune cell markers that hold significant value in the diagnosis and therapeutic monitoring of uveitis. For instance, measuring levels of IL-17 and IFN-γ can aid in assessing disease severity and treatment response. However, large-scale studies on immune cell types in human ocular diseases remain significantly limited.

GWAS conducted on large-scale samples have revealed some immune cells associated with uveitis from a genetic perspective. However, these studies have sample sizes sufficient only to identify major genetic influences. Factors limiting large-scale sample studies include difficulties in patient recruitment and international collaboration (9).

MR has emerged as a valuable technique in epidemiological studies for assessing causal relationships between exposures and outcomes. By leveraging genetic variations highly correlated with environmental factors as instrumental variables (10), MR utilizes genetic diversity, often represented by single nucleotide polymorphisms (SNPs), in molecular resonance. MR operates under the assumption that genetic variation is distributed randomly in the population, independent of environmental and confounding variables, effectively addressing concerns of reverse causation and bias from confounders (11). The advancement of MR has significantly facilitated the translation of GWAS findings into functionally relevant insights.

This study aims to investigate the relationship between 731 immune cells traits and uveitis risk by correlating large-scale GWAS data with immune cell traits using MR. Through this analysis, it seeks to elucidate the complex interplay among genetic susceptibility, immune inflammation, and uveitis. Ultimately, the research aims to enhance our understanding of the genetic basis of immune cell traits and uveitis.

## Methods

### Study design

**Figure 1:**
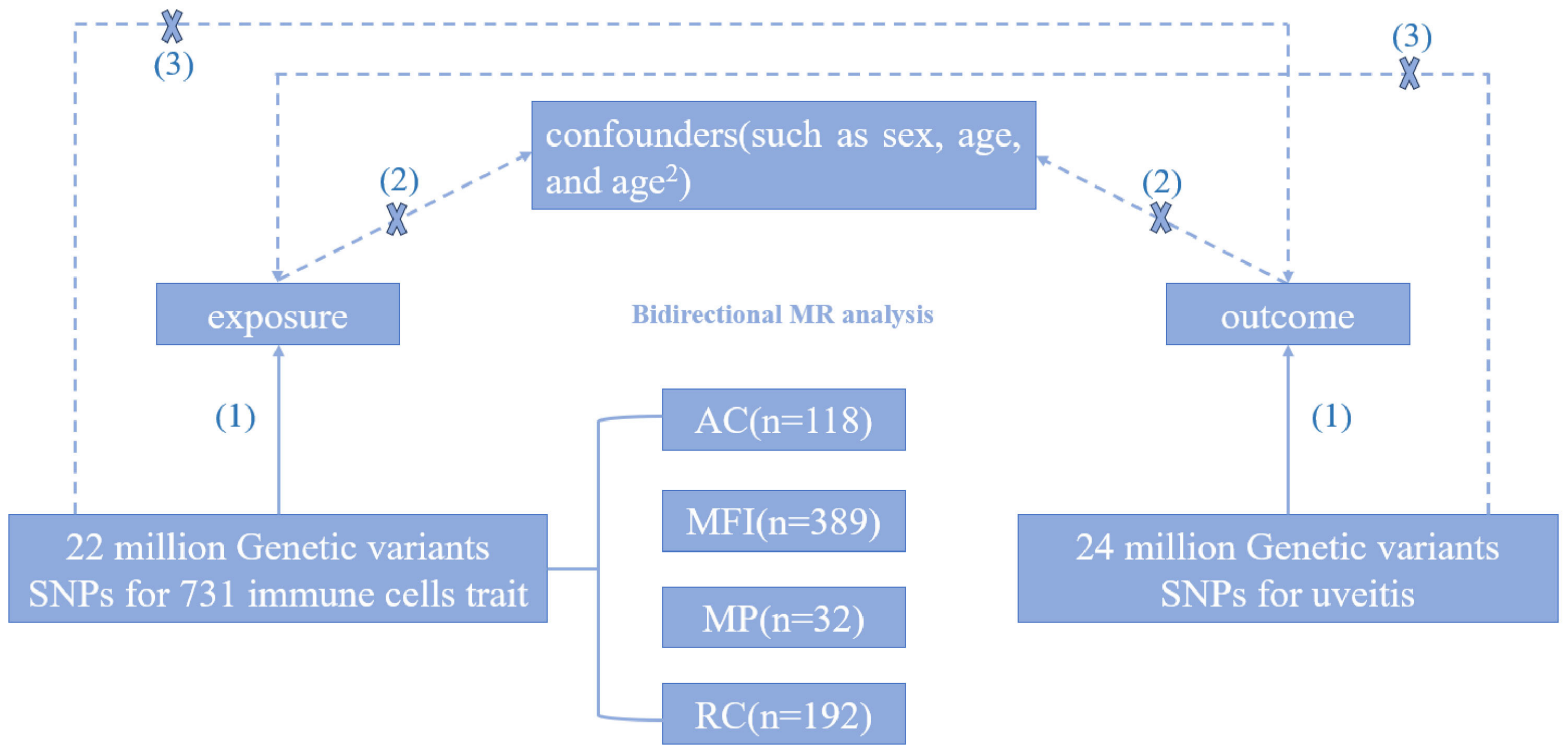
The design principles of MR study.

Through the two-sample Mendelian randomization analysis, we explored the causal relationship between 731 immune cells traits and uveitis. The flowchart of the study design is shown in **Figure 1**. In this study, Mendelian randomization analysis needs to satisfy the following three assumptions (1): Exposure is closely correlated with genetic variation (2); There is no correlation between genetic variation and confounding variables (3); Genetic variation affects outcomes only through exposure.

### Sources of immunity-wide GWAS data

Genome-wide association studies (GWAS) data for phenotypic profiles of 731 immunophenotypes were extracted from the GWAS catalog, spanning from GCST90001391 to GCST90002121 (12), involving a cohort of 3757 Europeans with no overlap across cohorts. Utilizing a high-density array comprising approximately 22 million SNPs, based on Sardinian sequence reference panels, correlations were examined while controlling for covariates such as age, age squared, and sex. The GWAS dataset encompassed absolute cell counts (AC) (n = 118), median fluorescence intensity (MFI) indicative of surface antigen level (n = 389), morphological parameter (MP) (n = 32), and relative cell counts (RC) (n = 192). Additionally, seven cell types were analyzed, including B cell maturation stage, cDCs maturation stage, T cell maturation stage, monocyte maturation stage, bone marrow cell maturation stage, TBNK (T cells, B cells, natural killer cells), and Treg maturation stage (**Supplementary Table 5**).

### Sources of uveitis GWAS data

The GWAS statistical data for uveitis were sourced from a study conducted by Sakaue et al. (13), utilizing diagnostic criteria based on ICD-10, with the disease code H-20. This study comprised a European population cohort consisting of 480,742 individuals, including 2,616 uveitis patients and 478,126 controls. Over 24 million SNPs were examined in this study to evaluate genetic associations with uveitis (**Supplementary Table 6**).

### Selection of instrumental variables

In order to guarantee the robustness of the instrumental variables, we employed stringent thresholds (*P* < 5 × 10^-8^) to identify SNPs that were significantly associated with presumed risk factors from published European genome-wide association studies (GWAS) (14). We then employed PLINK clustering to estimate the linkage imbalance between these SNPs for each risk factor, utilizing the 1000 genome European reference panel (15, 16). Finally, we employed MR analysis to incorporate SNPs that had the largest impact on immune traits based on chain imbalance, employing standards like r² > 0.001 and an aggregation window < 10,000 kb threshold (13). In the framework of a two-sample setup, we harmonized summary statistics and removed palindromes and incompatible alleles, thereby strengthening the dependability of our instrumental variable selection. According to GWAS Catalog, we removed SNPs associated with confounding factors [e.g. rheumatoid arthritis, inflammatory bowel disease, Crohn’s disease, ulcerative colitis, multiple sclerosis, systemic lupus erythematosus, psoriatic arthritis, ankylosing spondylitis, juvenile idiopathic arthritis, etc. (12)].

### Statistical analysis

The weighted median model (WMM), weighted median (WM), MR-Egger, and simple model were utilized to assess the robustness of the MR results. The IVW approach was our principal method for MR analysis. In addition, the heterogeneity in the MR analysis was tested using Cochrane’s Q technique. The MR Egger regression equation MR-PRESSO global test was utilized to thoroughly examine the presence of horizontal pleiotropy and P > 0.05 confirms the absence of horizontal pleiotropy (17). An assessment was made about the presence of a pleiotropy outlier (18). To test the robustness of the results of the MR analysis, we used the reservation-one method to exclude substantial effects of individual SNPs on the causal relationship between immunophenotypes and uveitis (19). Odds ratio (OR) indicates the influence of immunophenotypes on uveitis. P < 0.05 is the potential causal relationship. Reverse MR analysis is performed based on the causal relationship generated by forward MR analysis and summary statistics. The R software (version 4.3.2) and R package TwoSampleMR (version 0.5.6) was used for all analysis (20).

## Results

### Exploring the causal role of immunophenotypes on uveitis

To investigate the causal relationship between immunophenotypes and uveitis, we used two-sample MR analysis and excluded the effects of confounding factors such as some immune diseases. With a significance of 0.02, we found that 5 immunophenotypes were significantly associated with increased risk of uveitis, and 5 immunophenotypes were significantly associated with reduced risk of uveitis.

**Figure 2:**
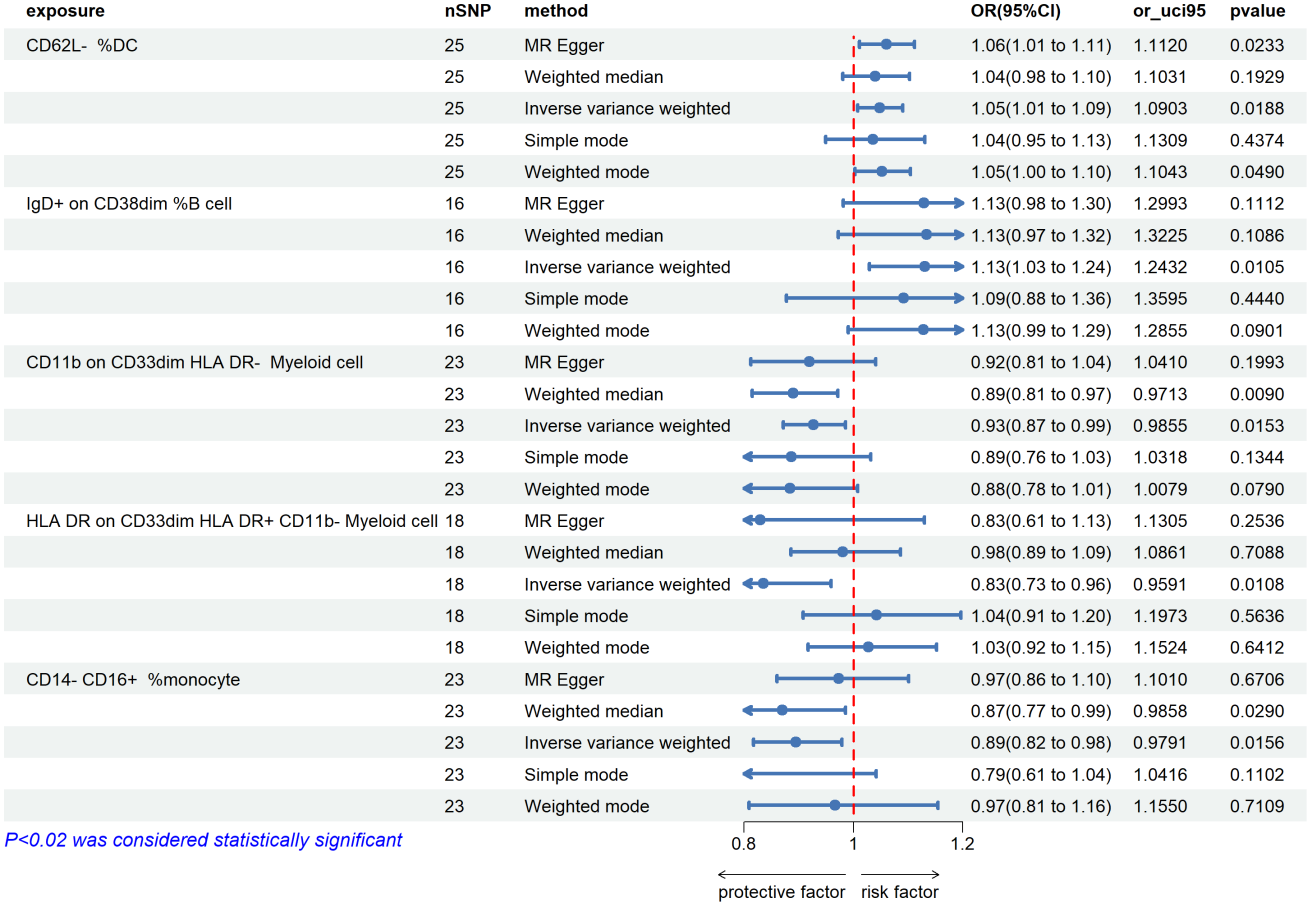
The forest plots showed a causal relationship between 5 immune cells traits and uveitis. MR, Mendelian randomization; SNP, single nucleotide polymorphism; CI, confidence interval.

**Figure 3:**
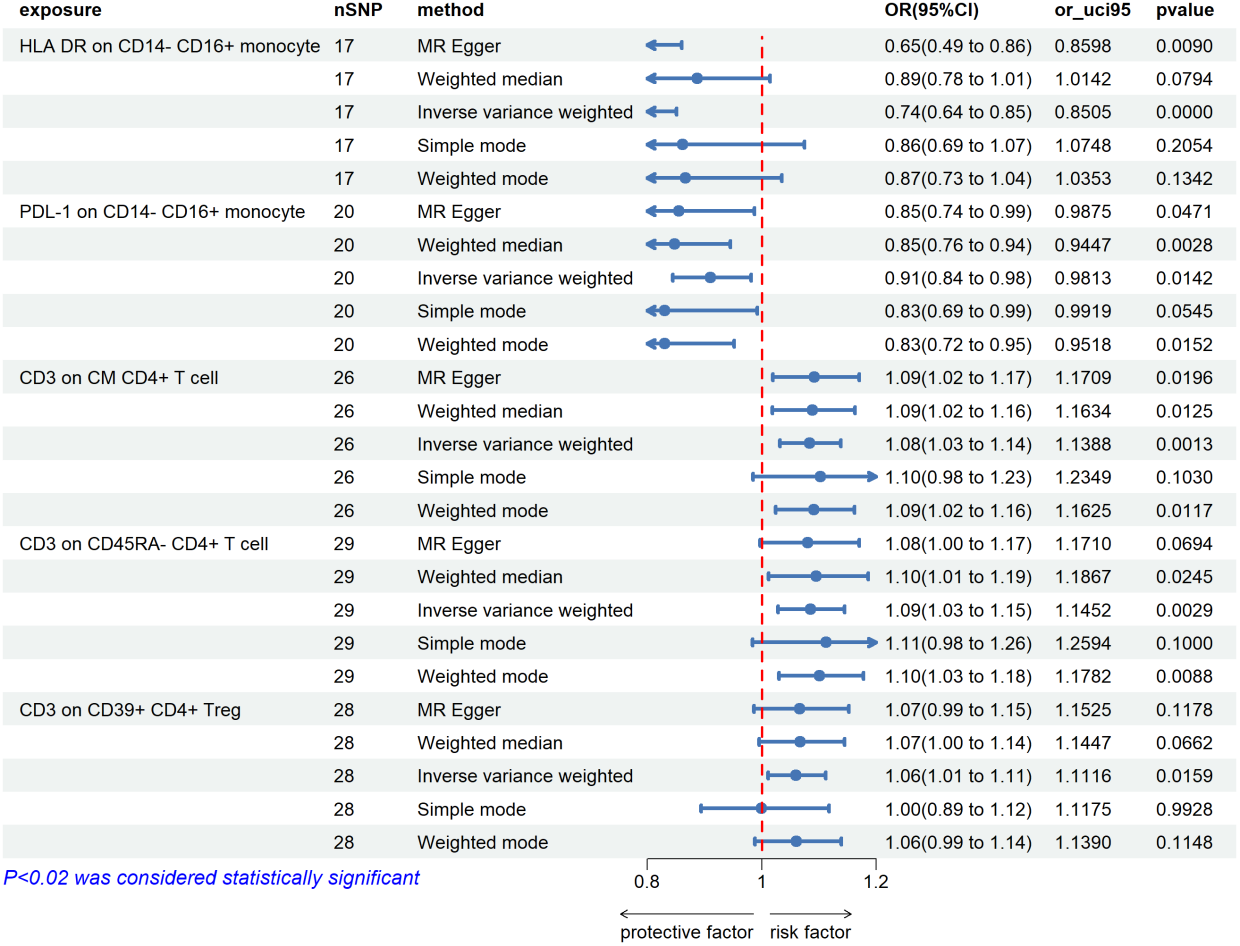
The forest plots showed a causal relationship between 5 immune cells traits and uveitis. MR, Mendelian randomization; SNP, single nucleotide polymorphism; CI, confidence interval.

By using the IVW method, we found 5 immunophenotypes associated with an increased risk of uveitis: CD62L-DC %DC (OR=1.045,95% CI=1.008-1.090, *P*=0.019), IgD+ CD38^dim^ %B cell (OR=1.131,95% CI=1.029-1.243, P=0.011) (**Figure 2**), CD3 on CM CD4+ T cells (OR=1.084,95% CI =1.032-1.13 = 0.0085, P=0.001), CD3 on CD45RA-CD4 +T cells (OR=1.085,95% CI=1.028-1.145, P=0.003), CD3 on CD39+ CD4+ Treg (OR=1.060,95% CI=1.011-1.112, P=0.016). (**Figure 3**). There are 5 immunophenotypes associated with a reduced risk of uveitis: CD11b on CD33^dim^ HLA DR-(OR= 0.927,95% CI= 0.871- 0.986, P=0.015), HLA DR on CD33^dim^ HLA DR+ CD11b- Myeloid cell (OR=0.835,95% CI=0.727-0.959, P=0.011), CD14-CD16+%monocyte (OR=0.895,95% CI=0.817-0.979, P=0.016) (**Figure 2**), HLA DR on CD14- CD16+ monocyte (OR=0.735,95% CI= 0.635 -0.851, P= 0.00), PDL-1 on CD14- CD16+ monocyte (OR=0.910,95% CI=0.844-0.981, P=0.014) (**Figure 3**).

The results of MR-Egger regression analysis, MR-PRESSO, and corrected distortion test showed no evidence of horizontal pleiotropy between immunophenotypes and uveitis (**Supplementary Tables 2**-**4**). Sensitivity analyses, as well as other analytical methods, indicated the strength of the causal association, while scatterplots and funnel plots indicated the robustness of the causal association (**Supplementary Figures 1**-**3**).

### Exploring the causal role of uveitis on immunophenotypes

For 10 types of immune cells that were significantly associated with uveitis, we performed reverse two-sample Mendelian randomization analysis, and there were 2 types of immune cells that still had significant reverse relationship with the risk of uveitis.

**Figure 4:**
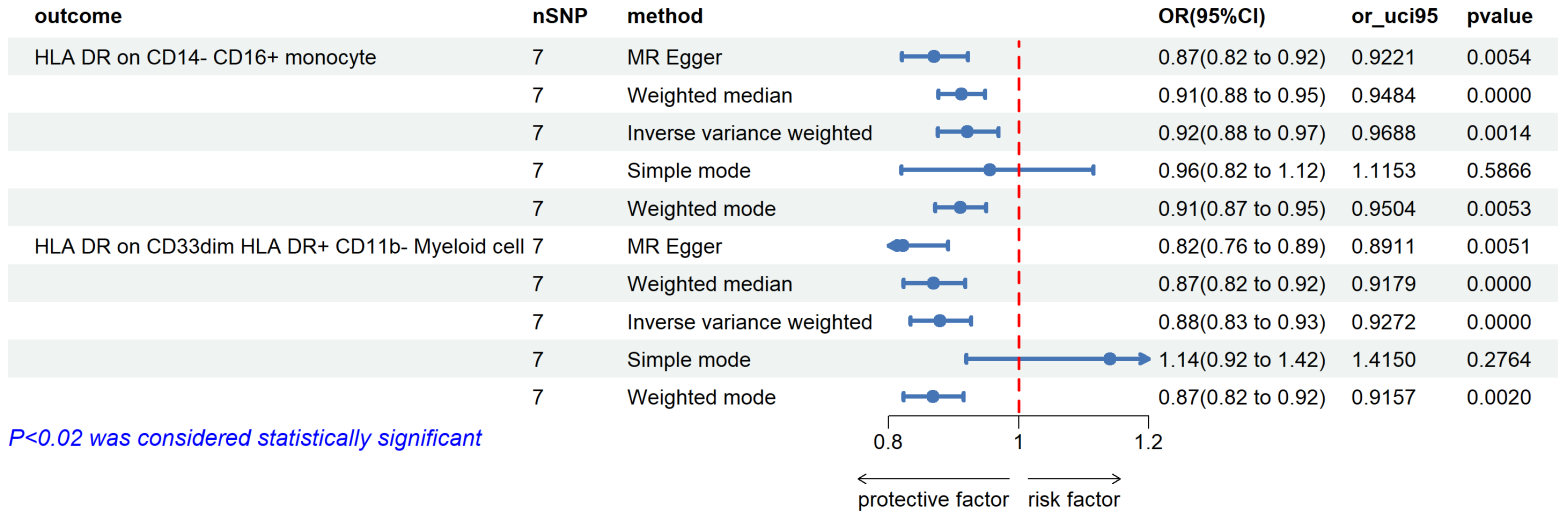
The forest plots showed a causal relationship between uveitis and immune cells traits. MR, Mendelian randomization; SNP, single nucleotide polymorphism; CI, confidence interval.

We found negative correlations in HLA DR on CD14-CD16 + monocyte and uveitis (OR=0.921, 95%CI =0.875-0.970, *P*=0.001). A similar association was also found in HLA DR on CD33^dim^ HLA DR+ CD11b- (OR=0.879, 95%CI = 0.833-0.927, *P*=0.00) (**Figure 4**).

There was no indication of horizontal pleiotropy between immunophenotypes and uveitis in the findings of the MR-Egger regression analysis, MR-PRESSO, or corrected distortion test (**Supplementary Table 2**). The robustness of the causal link was shown by scatterplots and funnel plots, whilst sensitivity analyses and other analytical techniques revealed the strength of the causal association (**Supplementary Figure 1**).

## Discussion

We explored the genetic link between immune cells and uveitis using extensive publicly available data. This pioneering study utilized MR analysis, marking the first of its kind in this context. Our analysis revealed 10 immune phenotypes significantly associated with uveitis – 5 linked to increased risk and 5 to decreased risk, 2 of them have a bi-directional relationship.

Immune surveillance stands as a critical function of the immune system, with migration ability being a key capability of dendritic cells (DCs) for this surveillance. Adhesion molecules play a pivotal role in facilitating DC migration (21). Specifically, CD62L facilitates the migration of DCs from infection sites to surrounding lymphoid tissues through interactions with sugar chains. The reduction in CD62L expression on DCs may therefore promote uveitis (22). A study of atrial fluid in patients with anterior uveitis showed that DCs were enriched at sites of inflammation in the AU, leading to subsequent proliferation and activation of effector T cells (23). Furthermore, an increase in the relative percentage of IgD+ CD38^dim^ B cells is positively correlated with uveitis risk. As noted in prior studies, the heightened production of plasma cells fosters inflammation by generating autoantibodies and via other mechanisms. Additionally, the expression level of CD38 on B cell surfaces impacts their functionality. CD38+ B cells (24), exhibiting elevated levels of ZAP-70 and heightened protein tyrosine phosphorylation, are associated with increased production of pro-inflammatory cytokines and suppression of CD4+ T cell proliferation, thus contributing to the pathogenesis of immune disorders (25).

Our study reveals that two phenotypes of bone marrow cells, characterized by CD11b on CD33^dim^ HLA DR- and HLA DR on CD33^dim^ HLA DR+ CD11b-, exhibit a negative association with uveitis risk. This observation may be attributed to their shared feature of expressing CD33. In recent years, a novel cell type known as myeloid-derived suppressor cells (MDSCs), possessing T-cell inhibitory activity, has emerged as a significant player in immune response regulation (26). Jeong et al. found that the number of MDSCs in the peripheral blood of patients with uveitis was significantly lower than that of healthy controls, and that they were highly increased during remission of the disease and progressively contracted upon cessation of the disease, which was used as a starting point for the study of MDSCs in a mouse model. They demonstrated that MDSCs expand in the spleen and mobilize into the bloodstream during peak retinal immune responses (27). Furthermore, they facilitate the spontaneous regression of inflammation, indicating the pivotal role of CD33+ myeloid cell in regulating autoimmune inflammation regression. This underscores their potential as a therapeutic target for uveitis treatment.

Non-classical monocytes (NCM) primarily contribute to vascular patrol and surveillance (28). In uveitis, particularly in cases associated with Behçet’s disease (BD), retinal vasculitis is a known pathological mechanism. A study investigating monocyte subpopulations in BD patients observed a reduced percentage of NCM, strongly correlated with disease activity, which normalized post-treatment. This finding underscores the potential association between decreased peripheral blood NCM and heightened vascular inflammation (29). Moreover, it suggests that NCM recruited to inflammatory sites may differentiate into M2 anti-inflammatory macrophages, contributing to tissue repair (30). We found that increased NCM characterized by CD14-CD16+, HLA DR on CD14-CD16+ and PDL-1 on CD14-CD16+ were associated with a decreased risk of uveitis, suggesting that an increase in NCM may be associated with enhanced vascular anti-inflammatory effects.

One of the currently recognized pathogenic mechanisms of uveitis is the crossing of the blood-retinal barrier by autoreactive CD4+ T cells, activation of local myeloid cells, recruitment of leukocytes including macrophages and monocytes, triggering tissue damage and angiogenesis, and disruption of immune regulation of intraocular tissues (31). The present study validates the idea from a genetic perspective that CD3 on CM CD4+ T cell and CD3 on CD45RA- CD4+T cell is associated with an increased risk of uveitis.

Based on a recent large-scale GWAS cohort study, this study is the first to investigate the causal relationship between 731 immune cells traits and uveitis from a genetic perspective. However, there are still limitations in our study. First, only GWAS data from European populations were selected for this study, which leads to the possibility that the results of this study may not be applicable to other ethnic populations. Additionally, the lack of validation from cohort studies is a notable limitation of our research. Given the exploratory nature of our study, subsequent cohort validation studies based on our findings could further address the current gap in understanding the relationship between immune cells and the risk of uveitis. Lastly, the original uveitis GWAS data did not provide detailed categorization of included patients (such as infectious and noninfectious classifications). As a result, our findings do not further elucidate the connection between the immune phenotypes of innate or adaptive immune cells and the origin of the disease. The publication of future large-scale GWAS studies with more comprehensive classifications may help rectify this limitation. In addition, the use of techniques such as fine-tuned localization can provide greater insight into how specific genetic variants affect immune cell function.

## Conclusion

In summary, our comprehensive MR analysis has demonstrated causal relationships between 11 immune cell phenotypes and uveitis in European populations. Specifically, CD62L- DC %DC, IgD+ CD38^dim^ %B cells, and CD3 expression on CM CD4+ T cells were associated with an increased risk of uveitis, while CD14- CD16+ monocyte %monocytes and CD11b expression on CD33^dim^ HLA DR- were associated with a decreased risk, and 2 of them were strongly associated with uveitis bi-directionally. This genetic perspective reveals the intricate connection between immune cell phenotypes and uveitis, offering new avenues for understanding the immunological mechanisms of uveitis and exploring novel immunotherapeutic approaches for its management.

## Data availability statement

The original contributions presented in the research are included in the article/**Supplementary Material**. Further inquiries can be directed to the corresponding authors.

## Ethics statement

The study protocols were approved by respective local ethics committees, and participants have provided written informed consent. The studies were conducted in accordance with the local legislation and institutional requirements. Written informed consent for participation was not required from the participants or the participants’ legal guardians/next of kin in accordance with the national legislation and institutional requirements.

## Author contributions

JW: Data curation, Formal analysis, Software, Writing – original draft. CF: Methodology, Writing – original draft. YZ: Formal analysis, Writing – original draft. MW: Funding acquisition, Writing – original draft. QL: Funding acquisition, Writing – review & editing. SD: Funding acquisition, Writing – review & editing.
