# Supplementary figures and images for: Causal role of immune cells in uveitis: Mendelian randomization study
Source: Front Immunol. 2024 Jul 9;15:1402074. doi: 10.3389/fimmu.2024.1402074 (PMC11263026; doi:10.3389/fimmu.2024.1402074)

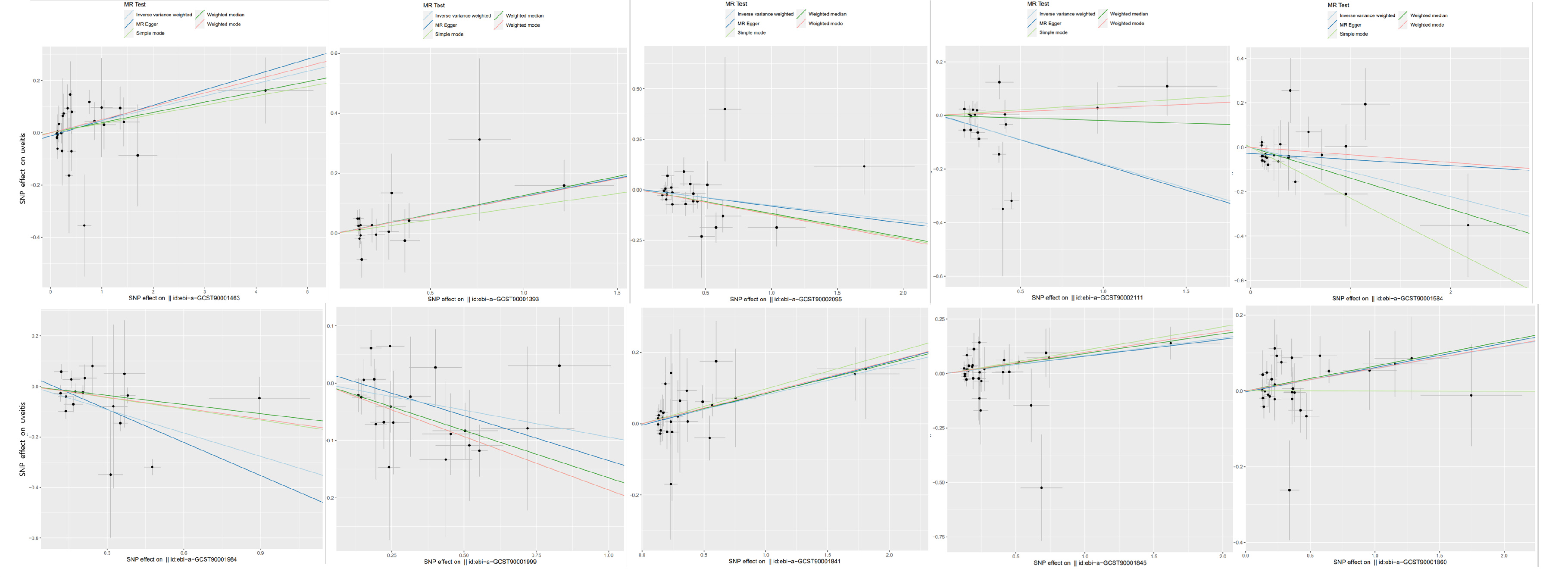

Supplement: Supplementary file 1 [file DataSheet_1.zip › supplementary-revision V3.0/Supplementary figure 1.tif]

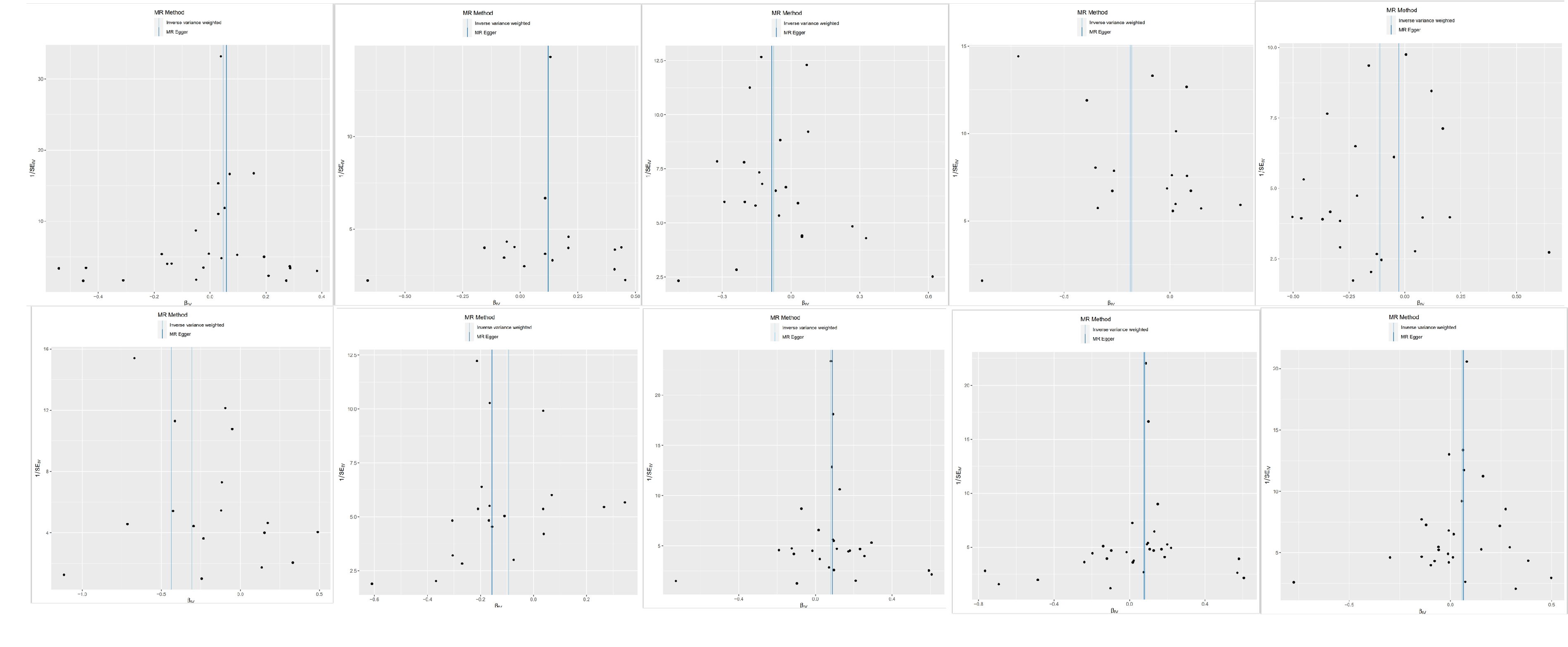

Supplement: Supplementary file 1 [file DataSheet_1.zip › supplementary-revision V3.0/Supplementary figure 2.tif]

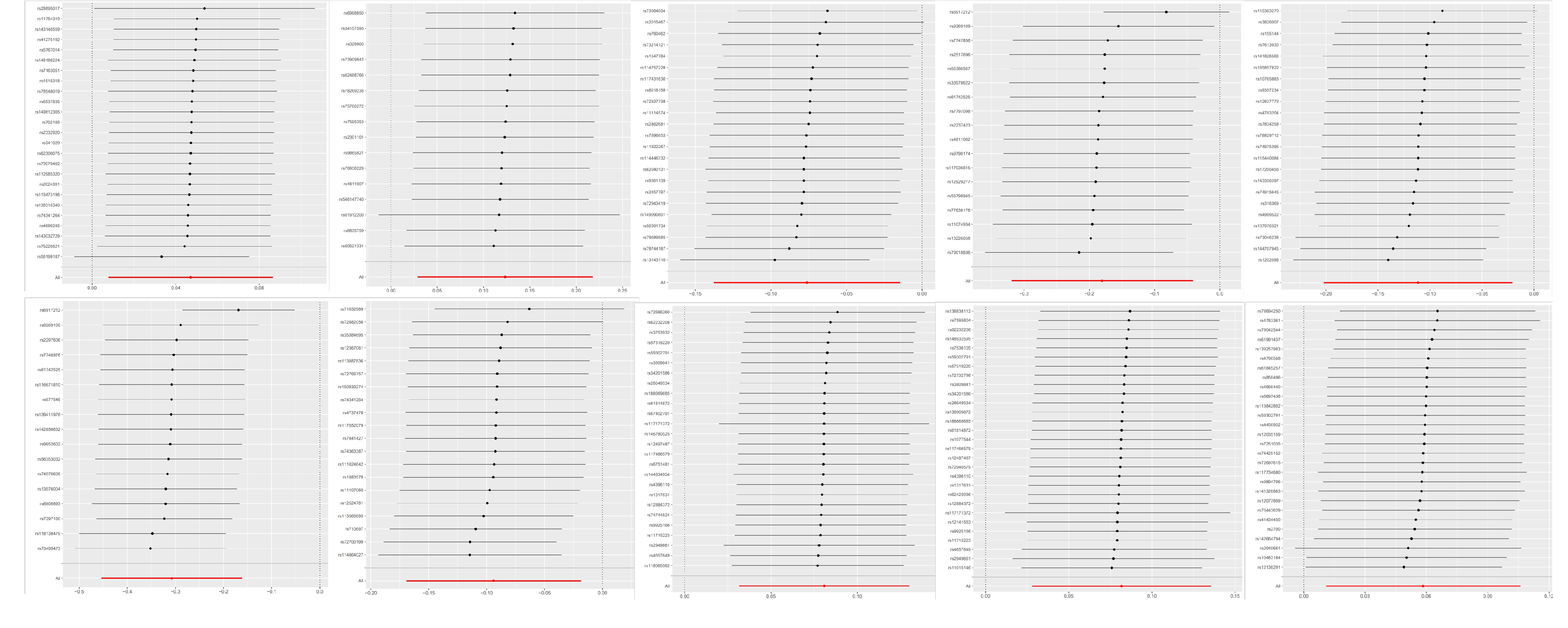

Supplement: Supplementary file 1 [file DataSheet_1.zip › supplementary-revision V3.0/Supplementary figure 3.tif]
