# Supplementary material for: Causal role of immune cells in uveitis: Mendelian randomization study
Source: Front Immunol. 2024 Jul 9;15:1402074. doi: 10.3389/fimmu.2024.1402074 (PMC11263026; doi:10.3389/fimmu.2024.1402074)
Supplement: Supplementary file 2 [file DataSheet_2.pdf]

STROBE-MR checklist of recommended items to address in reports of Mendelian randomization studies

Item No.	Section	Checklist item	Relevant text from manuscript
1	**TITLE and ABSTRACT**	Indicate Mendelian randomization (MR) as the study's design in the title and/or the abstract if that is a main purpose of the study	Causal role of immune cells in uveitis: Mendelian randomization study
	**INTRODUCTION**		
2	**Background**	Explain the scientific background and rationale for the reported study. What is the exposure? Is a potential causal relationship between exposure and outcome plausible? Justify why MR is a helpful method to address the study question	Uveitis, characterized by inflammation of the iris, ciliary body, and choroid, presents a significant global clinical challenge, contributing substantially to visual impairment. Risk factors include autoimmune diseases and immune cell dysfunctions, yet many remain unidentified. Immune cells, notably T cells, B cells, and monocytes, play pivotal roles in uveitis pathogenesis. While biologic agents show promise, comprehensive studies on immune cell types in ocular diseases are lacking. Genome-wide association studies (GWAS) and Mendelian randomization (MR) present promising avenues to elucidate genetic susceptibilities and causal relationships between immune cell traits and uveitis risk.
3	**Objectives**	State specific objectives clearly, including pre-specified causal hypotheses (if any). State that MR is a method that, under specific assumptions, intends to estimate causal effects	This study aims to thoroughly investigate the causal relationship between 731 types of immune cells and uveitis. It seeks to uncover immune cells not previously associated with uveitis using the Mendelian randomization (MR) method, while also confirming the involvement of immune cells already linked to the condition. By doing so, the research aims to elucidate the complex relationship between genetic susceptibility, immune inflammation, and genetic background in uveitis patients, thereby enhancing our understanding of its underlying genetic factors.
	**METHODS**		
4	**Study design and data sources**	Present key elements of the study design early in the article. Consider including a table listing sources of data for all phases of the study. For each data source contributing to the analysis, describe the following:	

		a)	Setting: Describe the study design and the underlying population, if possible. Describe the setting, locations, and relevant dates, including periods of recruitment, exposure, follow-up, and data collection, when available.	Through the two-sample Mendelian randomization analysis, we explored the bidirectional causal relationship between 731 immune cells traits and uveitis. The flowchart of the study design is shown in Figure 1.
		b)	Participants: Give the eligibility criteria, and the sources and methods of selection of participants. Report the sample size, and whether any power or sample size calculations were carried out prior to the main analysis	GWAS statistics for uveitis were sourced from a study conducted by Sakaue et al.(1), encompassing a European population cohort comprising 480,742 individuals, of which 2,616 were patients with uveitis and 478,126 served as controls. The study examined over 24 million SNPs to assess genetic associations with uveitis.
		c)	Describe measurement, quality control and selection of genetic variants	We rigorously selected SNPs associated with risk factors from European GWAS (P< 5 × 10-8). Using PLINK clustering and MR Analysis, we assessed linkage imbalance and prioritized SNPs with significant impact on immune traits ($r^2 > 0.001$, aggregation window < 10,000 kb). Harmonizing summary statistics and removing palindromes and incompatible alleles in a two-sample setup strengthened our instrumental variable selection's reliability. According to GWAS Catalog, we removed SNPs associated with confounding factors.
		d)	For each exposure, outcome, and other relevant variables, describe methods of assessment and diagnostic criteria for diseases	For uveitis, diagnostic criteria based on ICD-10, with the disease code H-20. For immune cells traits, the GWAS data came from the GWAS catalog, spanning from GCST90001391 to GCST90002121(2).
		e)	Provide details of ethics committee approval and participant informed consent, if relevant	N/A
5	**Assumptions**		Explicitly state the three core IV assumptions for the main analysis (relevance, independence and exclusion restriction) as well assumptions for any additional or sensitivity analysis	In this study, Mendelian randomization analysis needs to satisfy the following three assumptions: (1) Exposure is closely correlated with genetic variation;(2) There is no correlation between genetic variation and confounding variables;(3) Genetic variation affects outcomes only through exposure.
6	**Statistical methods: main analysis**		Describe statistical methods and statistics used	

		a)	Describe how quantitative variables were handled in the analyses (i.e., scale, units, model)	The weighted median model (WMM), weighted median (WM), MR-Egger, and simple model were utilized to assess the robustness of the MR results. The IVW approach was our principal method for MR analysis.
		b)	Describe how genetic variants were handled in the analyses and, if applicable, how their weights were selected	In order to guarantee the robustness of the instrumental variables, we employed stringent thresholds ($P < 5 \times 10^{-8}$) to identify SNPS that were significantly associated with presumed risk factors from published European genome-wide association studies (GWAS). We then employed PLINK clustering to estimate the linkage imbalance between these SNPS for each risk factor, utilizing the 1000 genome European reference panel. Finally, we employed MR Analysis to incorporate SNPS that had the largest impact on immune traits based on chain imbalance, employing standards like $r^2 > 0.001$ and an aggregation window < 10,000 kb threshold. In the framework of a two-sample setup, we harmonized summary statistics and removed palindromes and incompatible alleles, thereby strengthening the dependability of our instrumental variable selection. According to GWAS Catalog, we removed SNPs associated with confounding factors.
		c)	Describe the MR estimator (e.g. two-stage least squares, Wald ratio) and related statistics. Detail the included covariates and, in case of two-sample MR, whether the same covariate set was used for adjustment in the two samples	Odds ratio (OR) indicates the influence of various immune cells on uveitis. $P < 0.02$ is the significant causal relationship. Reverse MR analysis is performed based on the causal relationship generated by forward MR analysis and summary statistics.
		d)	Explain how missing data were addressed	N/A
		e)	If applicable, indicate how multiple testing was addressed	N/A
7	**Assessment of assumptions**		Describe any methods or prior knowledge used to assess the assumptions or justify their validity	The MR Egger regression equation MR-PRESSO global test was utilized to thoroughly examine the presence of horizontal pleiotropy and $P > 0.05$ confirms the absence of horizontal pleiotropy. An assessment was made about the presence of a pleiotropy outlier.
8	**Sensitivity analyses and**		Describe any sensitivity analyses or additional analyses performed (e.g. comparison of effect estimates from different approaches, independent replication, bias analytic techniques, validation of instruments, simulations)	In addition, the heterogeneity in the MR analysis was tested using Cochrane's Q technique. To test the robustness of the results of the MR analysis, we used the leave-one-out method to exclude

	**additional analyses**			substantial effects of individual SNPs on the causal relationship between immune cells traits and uveitis.
9	**Software and pre-registration**			
		a)	Name statistical software and package(s), including version and settings used	The R software (version 4.3.2) and R package TwoSampleMR (version 0.5.6) was used for all analysis.
		b)	State whether the study protocol and details were pre-registered (as well as when and where)	None.
	**RESULTS**			
10	**Descriptive data**			
		a)	Report the numbers of individuals at each stage of included studies and reasons for exclusion. Consider use of a flow diagram	To investigate the causal relationship between immunophenotype and uveitis, we used two-sample MR analysis ($P < 0.05$). With a significance of 0.02, we found that 5 types of immune cells were significantly associated with increased risk of uveitis, 5 types of immune cells were significantly associated with reduced risk of uveitis, and 2 of them have a bi-directional relationship.
		b)	Report summary statistics for phenotypic exposure(s), outcome(s), and other relevant variables (e.g. means, SDs, proportions)	N/A
		c)	If the data sources include meta-analyses of previous studies, provide the assessments of heterogeneity across these studies	N/A
		d)	For two-sample MR:<br>i. Provide justification of the similarity of the genetic variant-exposure associations between the exposure and outcome samples<br>ii. Provide information on the number of individuals who overlap between the exposure and outcome studies	i In order to guarantee the robustness of the instrumental variables, we employed stringent thresholds ($P < 5 \times 10^{-8}$) to identify SNPS that were significantly associated with presumed risk factors from published European genome-wide association studies (GWAS).<br>ii None overlapping cohorts.
11	**Main results**			
		a)	Report the associations between genetic variant and exposure, and between genetic variant and outcome, preferably on an interpretable scale	To investigate the causal relationship between immunophenotype and uveitis, we used two-sample MR Analysis and FDR correction ($P < 0.05$). With a significance of 0.02, we found that 5 types of immune cells were significantly associated with increased risk of uveitis, 5 types of immune cells

			were significantly associated with reduced risk of uveitis, and 2 of them have a bi-directional relationship.
		b) Report MR estimates of the relationship between exposure and outcome, and the measures of uncertainty from the MR analysis, on an interpretable scale, such as odds ratio or relative risk per SD difference	By using the IVW method, we found 5 immunophenotypes associated with an increased risk of uveitis: CD62L-DC %DC (OR=1.045,95% CI=1.008-1.090, *P*=0.019), IgD+ CD38^{dim} %B cell (OR=1.131,95% CI=1.029-1.243, *P*=0.011) (*Figure 2*), CD3 on CM CD4+ T cells (OR=1.084,95% CI =1.032-1.13=0.0085, *P*=0.001), CD3 on CD45RA- CD4 +T cells (OR=1.085,95% CI=1.028-1.145, *P*=0.003), CD3 on CD39+ CD4+ Treg (OR=1.060,95% CI=1.011-1.112, *P*=0.016). (*Figure 3*). There are 5 immunophenotypes associated with a reduced risk of uveitis: CD11b on CD33^{dim} HLA DR-(OR= 0.927,95% CI= 0.871- 0.986, *P*=0.015), HLA DR on CD33^{dim} HLA DR+ CD11b- Myeloid cell (OR=0.835,95% CI=0.727-0.959, *P*=0.011), CD14- CD16+%monocyte (OR=0.895,95% CI=0.817-0.979, *P*=0.016) (*Figure. 2*), HLA DR on CD14- CD16+ monocyte (OR=0.735,95% CI= 0.635 -0.851, *P*= 0.00), PDL-1 on CD14- CD16+ monocyte (OR=0.910,95% CI=0.844-0.981, *P*=0.014) (*Figure 3*).<br><br>We found negative correlations in HLA DR on CD14- CD16 + monocyte and uveitis (OR=0.921, 95%CI =0.875-0.970, *P*=0.001). A similar association was also found in HLA DR on CD33^{dim} HLA DR+ CD11b- (OR=0.879, 95%CI = 0.833-0.927, *P*=0.00) (*Figure 4*).
		c) If relevant, consider translating estimates of relative risk into absolute risk for a meaningful time period	N/A
		d) Consider plots to visualize results (e.g. forest plot, scatterplot of associations between genetic variants and outcome versus between genetic variants and exposure)	Supplementary figure 1-3
12	**Assessment of assumptions**		
		a) Report the assessment of the validity of the assumptions	The results of MR-Egger regression analysis, MR-PRESSO, and corrected distortion test showed no evidence of horizontal pleiotropy between immunophenotypes and uveitis (Supplementary Table 2 and 4).

		b)	Report any additional statistics (e.g., assessments of heterogeneity across genetic variants, such as I^2, Q statistic or E-value)	Supplementary table 3
13	**Sensitivity analyses and additional analyses**			
		a)	Report any sensitivity analyses to assess the robustness of the main results to violations of the assumptions	Sensitivity analyses, as well as other analytical methods, indicated the strength of the causal association, while scatterplots and funnel plots indicated the robustness of the causal association (*Supplementary Figure 1*).
		b)	Report results from other sensitivity analyses or additional analyses	Supplementary table 2 and 4
		c)	Report any assessment of direction of causal relationship (e.g., bidirectional MR)	For 10 types of immune cells that were significantly associated with uveitis, and 2 of them have a bi-directional relationship.
		d)	When relevant, report and compare with estimates from non-MR analyses	N/A
		e)	Consider additional plots to visualize results (e.g., leave-one-out analyses)	Supplementary figure 3
	**DISCUSSION**			
14	**Key results**		Summarize key results with reference to study objectives	We explored the genetic link between immune cells and uveitis using extensive publicly available data. This pioneering study utilized MR analysis, marking the first of its kind in this context. Our analysis revealed 10 immune phenotypes significantly associated with uveitis – 5 linked to increased risk and 5 to decreased risk, 2 of them have a bi-directional relationship.
15	**Limitations**		Discuss limitations of the study, taking into account the validity of the IV assumptions, other sources of potential bias, and imprecision. Discuss both direction and magnitude of any potential bias and any efforts to address them	First, only GWAS data from European populations were selected for this study, which leads to the possibility that the results of this study may not be applicable to other ethnic populations. Additionally, the lack of validation from cohort studies is a notable limitation of our research. Given the exploratory nature of our study, subsequent cohort validation studies based on our findings could further address the current gap in understanding the relationship between immune cells and the risk of uveitis. Lastly, the original uveitis GWAS data did not provide detailed categorization of included patients (such as infectious and noninfectious classifications). As a

			result, our findings do not further elucidate the connection between the immune phenotypes of innate or adaptive immune cells and the origin of the disease. The publication of future large-scale GWAS studies with more comprehensive classifications may help rectify this limitation. In addition, the use of techniques such as fine-tuned localization can provide greater insight into how specific genetic variants affect immune cell function.
16	**Interpretation**		
		a) Meaning: Give a cautious overall interpretation of results in the context of their limitations and in comparison with other studies	Based on a recent large-scale GWAS cohort study, this study is the first to investigate the bidirectional causal relationship between 731 immune cell phenotypes and uveitis from a genetic perspective.
		b) Mechanism: Discuss underlying biological mechanisms that could drive a potential causal relationship between the investigated exposure and the outcome, and whether the gene-environment equivalence assumption is reasonable. Use causal language carefully, clarifying that IV estimates may provide causal effects only under certain assumptions	Dendritic cells (DCs) play a vital role in immune surveillance due to their migration ability facilitated by adhesion molecules like CD62L. Reduced CD62L expression on DCs may promote uveitis. Higher IgD+ CD38dim B cell percentages correlate with uveitis risk, leading to increased plasma cell production and inflammation.
		c) Clinical relevance: Discuss whether the results have clinical or public policy relevance, and to what extent they inform effect sizes of possible interventions	Non-classical monocytes (NCM) primarily contribute to vascular patrol and surveillance. In uveitis, particularly in cases associated with Behçet's disease (BD), retinal vasculitis is a known pathological mechanism. A study investigating monocyte subpopulations in BD patients observed a reduced percentage of NCM, strongly correlated with disease activity, which normalized post-treatment. This finding underscores the potential association between decreased peripheral blood NCM and heightened vascular inflammation. Moreover, it suggests that NCM recruited to inflammatory sites may differentiate into M2 anti-inflammatory macrophages, contributing to tissue repair. We found that increased NCM characterized by CD14-CD16+, HLA DR on CD14-CD16+ and PDL-1 on CD14-CD16+ were associated with a decreased risk of uveitis, suggesting that an increase in NCM may be associated with enhanced vascular anti-inflammatory effects
17	**Generalizability**	Discuss the generalizability of the study results (a) to other populations, (b) across other exposure periods/timings, and (c) across other levels of exposure	N/A

	**OTHER INFORMATION**		
18	**Funding**	Describe sources of funding and the role of funders in the present study and, if applicable, sources of funding for the databases and original study or studies on which the present study is based	This study was supported by Medical Science and Technology Project (the Key Project Jointly Built by the Province and the Ministry) of Henan Province (no. SBGJ202102167 and LHGJ20190187).
19	**Data and data sharing**	Provide the data used to perform all analyses or report where and how the data can be accessed, and reference these sources in the article. Provide the statistical code needed to reproduce the results in the article, or report whether the code is publicly accessible and if so, where	The original contributions presented in the research are included in the article/supplementary material. Further inquiries can be directed to the corresponding author.
20	**Conflicts of Interest**	All authors should declare all potential conflicts of interest	All authors declare to have no conflict of interest.

This checklist is copyrighted by the Equator Network under the Creative Commons Attribution 3.0 Unported (CC BY 3.0) license.

1. Sakaue S, Kanai M, Tanigawa Y, Karjalainen J, Kurki M, Koshiba S, et al. A Cross-Population Atlas of Genetic Associations for 220 Human Phenotypes. *Nature genetics* (2021) 53(10):1415-24. Epub 2021/10/02. doi: 10.1038/s41588-021-00931-x.
2. Orrù V, Steri M, Sidore C, Marongiu M, Serra V, Olla S, et al. Complex Genetic Signatures in Immune Cells Underlie Autoimmunity and Inform Therapy. *Nature genetics* (2020) 52(10):1036-45. Epub 2020/09/16. doi: 10.1038/s41588-020-0684-4.
